# Effects of enriched task-specific training on sit-to-stand tasks in individuals with chronic stroke

**DOI:** 10.3233/NRE-230204

**Published:** 2024-03-11

**Authors:** Sara Vive, Roland Zügner, Roy Tranberg, Lina Bunketorp-Käll

**Affiliations:** a Section for Health and Rehabilitation, Institute of Neuroscience and Physiology, Sahlgrenska Academy, University of Gothenburg, Gothenburg, Sweden; b Neurocampus, Sophiahemmet Hospital, Stockholm, Sweden; c Department of Orthopedics, Institute of Clinical Sciences, Sahlgrenska Academy, University of Gothenburg, Gothenburg, Sweden; d Centre for Advanced Reconstruction of Extremities (CARE), Sahlgrenska University Hospital, Mölndal, Sweden

**Keywords:** Enriched environment, movement analysis, recovery of function, rehabilitation, sit-to-stand, stroke

## Abstract

**BACKGROUND:**

Approximately 80% of stroke survivors experience motor impairment of the contralateral limb that severely affects their activities of daily living (ADL).

**OBJECTIVE:**

To evaluate whether an enriched task-specific training (ETT) program affected the performance and kinetics of sit-to-stand (STS) tasks.

**METHODS:**

The study was part of an exploratory study with a within-subject, repeated-measure-design, with assessments before and after a three-week-long baseline period, and six months after the intervention. Forty-one participants underwent assessments of strength and endurance measured by the 30-second-chair-stand test (30sCST). The STS-kinetics, including the vertical ground reaction force (GRF) during STS, were analysed in an in-depth-subgroup of three participants, using a single-subject-experimental-design (SSED). For kinetic data, statistical significance was determined with the two-standard deviation band method (TSDB).

**RESULTS:**

After the baseline period, a small increase was seen in the 30sCST (from 5.6±4.5 to 6.1±4.9, *p* = 0.042). A noticeable significant change in the 30sCST was shown after the intervention (from 6.1±4.9 to 8.2±5.4, *p* < 0.001), maintained at six months. The in-depth kinetic analyses showed that one of three subjects had a significant increase in loading of the affected limb post-intervention.

**CONCLUSION:**

ETT can produce long-term gains in STS performance. Weight-bearing strategies could be one of several factors that contribute to improvements in STS performance in the chronic phase after stroke.

## 1 Introduction

Approximately 80% of stroke survivors experience motor impairment of the contralateral limb— i.e., hemiparesis— that severely affects their activities of daily living (ADL). Moreover, one-third of individuals suffer from persistent impairments 6– 12 months after stroke onset (Asplund et al., 2003) that largely affect independence, quality of life (QoL), and participation (Duncan et al., 2005; Langhorne, Bernhardt, & Kwakkel, 2011; Collaborators, 2019). Many of those who recover the ability to walk after stroke retain a motor deficit characterized by marked asymmetry, poor postural control, and limited endurance. Stroke-related asymmetry causes impairment in the achievement of functional tasks and voluntary movements. The asymmetrical nature of functional tasks is characterized by reduced weight-bearing on the affected lower limb in comparison with the non-affected side (Gray & Culham, 2014). Impaired motor function and weight-bearing asymmetry may contribute to increased fall risk during transition movements, including sit-to-stand (STS) movement. Consequently, improved weight-bearing symmetry and balance during STS movement are goals of rehabilitation in this population. However, research in stroke rehabilitation does not always succeed in distinguishing whether a change in performance reflects true recovery or compensation (Krakauer, 2006; Levin, Kleim, & Wolf, 2009; Bernhardt et al., 2017). Treatment-induced improvements may be related to brain plasticity, restorative/healing processes, and/or compensatory mechanisms (Levin et al., 2009; Bernhardt et al., 2017). True recovery defines restoring the ability to perform a movement in the same kinematic manner as it was performed before the injury. Compensation is described as performing an old movement in a new manner, using alternative movement pattern, resulting in a change in muscle activation as well as different timing and kinematic patterns (Krakauer, 2006; Levin et al., 2009; Bernhardt et al., 2017).

In biomechanical terms, STS movement can be defined as a transitional movement into the upright posture requiring movement of the center of mass from a lower stable position into a more unstable up right position with expected extended hip and knee joints together with ankle joints in a neutral (0°) position (Vander Linden, Brunt, & McCulloch, 1994). The dynamic of STS movement is usually described using kinematic and kinetic variables. Kinematic analysis allows the investigator to study the spatial and temporal characteristics of the STS movement, whilst kinetic analysis concerns the forces created by and during the movement.

The achievement strategy for the STS movement differs considerably with the subject’s intent on the movement (Yamada & Demura, 2005). The STS movement strategy during test situations most likely depends on whether the subject performs a self-administered movement or receives specific instructions on what strategy to use when rising from the seated position. Various therapeutic approaches are used with the aim to improve STS capacity in individuals with stroke (Barreca, Sigouin, Lambert, & Ansley, 2004; Britton, Harris, & Turton, 2008; Malouin, Richards, Durand, & Doyon, 2009). Previous studies suggest that therapies based on the principle of task-oriented exercise can significantly improve the motor and functional abilities of the upper and lower limbs (Veerbeek et al., 2014). We have previously reported that a rehabilitation program consisting of enriched task-specific training (ETT) provides durable benefits across a wide spectrum of motor deficits and impairments after stroke (S. Vive, Bunketorp-Kall, & Carlsson, 2020; S. Vive, Af Geijerstam, Kuhn, & Bunketorp-Kall, 2020; S Vive, Zugner, Tranberg, & Bunketorp-Kall, 2021). It is yet unknown whether ETT produces gains in STS performance. We therefore conducted secondary analyses on existing data to explore whether the ETT intervention impacted on the performance and kinetic features of STS tasks in individuals with chronic stroke. More specifically, the first aim was to examine the influence of ETT on lower extremity strength and endurance as measured by an STS task. The second aim was to conduct in-depth assessments of weight-bearing asymmetry during an STS task using kinetic data and selected measures from the magnitude of the vertical ground reaction force (GRF) variables in both affected and non-affected limbs before and after the ETT program. An additional aim was to examine how the loading of the affected lower limb during an STS task was influenced when administering the test with and without specific instructions.

## 2 Methods

### 2.1 Study design and participants

**Fig. 1:**
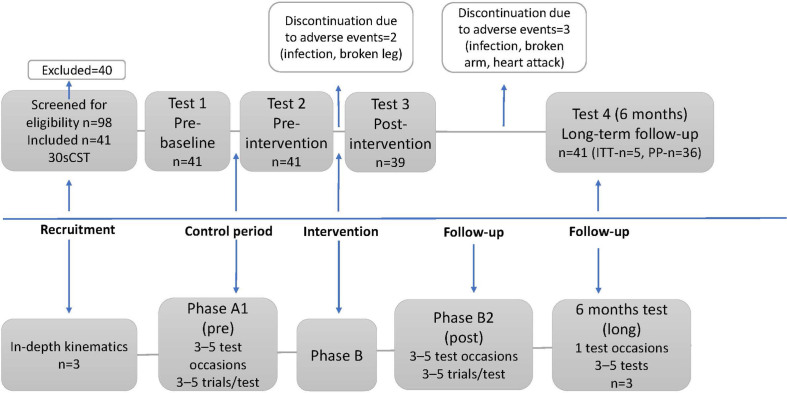
Flowchart of the study.

This study uses data from a previous exploratory study in which 41 individuals with chronic stroke were enrolled. The former study had a within-subject, repeated-measure design with assessment before and after a three-week-long baseline period as a control period, immediately after the intervention, and follow-up at six months. The eligibility criteria are listed in Table 1. For the in-depth analyses of kinetic data, a single-subject experimental design (SSED) was used (Barlow & Hersen, 1973; S Vive et al., 2021). In the SSED study, a subgroup of three ETT participants underwent three assessment phases (ABA). The initial phase (A1) was represented by the three-week baseline period before the ETT program. During this phase, data was collected on three to five occasions, at least one day apart. Three to five STS trials were performed on each measurement occasion (Zhan & Ottenbacher, 2001). The subjects were allowed to continue their regular treatment for a maximum of three hours per week during the baseline (A1) period but were asked not to start any new rehabilitation. The baseline period was followed by the three-week intervention period (B). Immediately after the intervention, Phase A was repeated (A2). A single follow-up measurement was done six months after completion of the ETT program. The flowchart of the study is presented in Fig. 1.

**Table 1:** Eligibility criteria

Eligibility criteria
At least 6 months and a maximum 10 years after the onset of stroke
Disability grade 2– 4 on the Modified Rankin Scale^a^
Baseline motor deficit defined as less than a full score on the primary outcome measure (M-MAS UAS^b^)
No other injury, illness, making the individual unsuitable for participation, including exercise-induced epilepsy, assessed by the referring or prescribing physician.
Cognitive and speech ability that enables instruction, intervention, and evaluation
Ability and willingness to travel to the place of evaluation
Able to perform sit-to-stand and stand-to-sit transfers independently or with assistance, without assistive technology such as mechanical lifts
Not having participated in a similar high-dose rehabilitation program (other than post-stroke acute and subacute rehabilitation) within the previous 6 months
Not scheduled for other treatment focused on intensive high-dose training during the study period
Expanded eligibility criteria for the kinetic part of the study
Live near Gothenburg, able to do repetitive measures in the gait lab according to the ABA design

^a^An ordinal disability rating scale, scored 0– 6 (0 = no symptoms). ^b^Modified Motor Assessment Scale developed at Uppsala University Hospital in 1999.

### 2.2 Intervention— Enriched task-specific therapy (ETT)

**Fig. 2:**
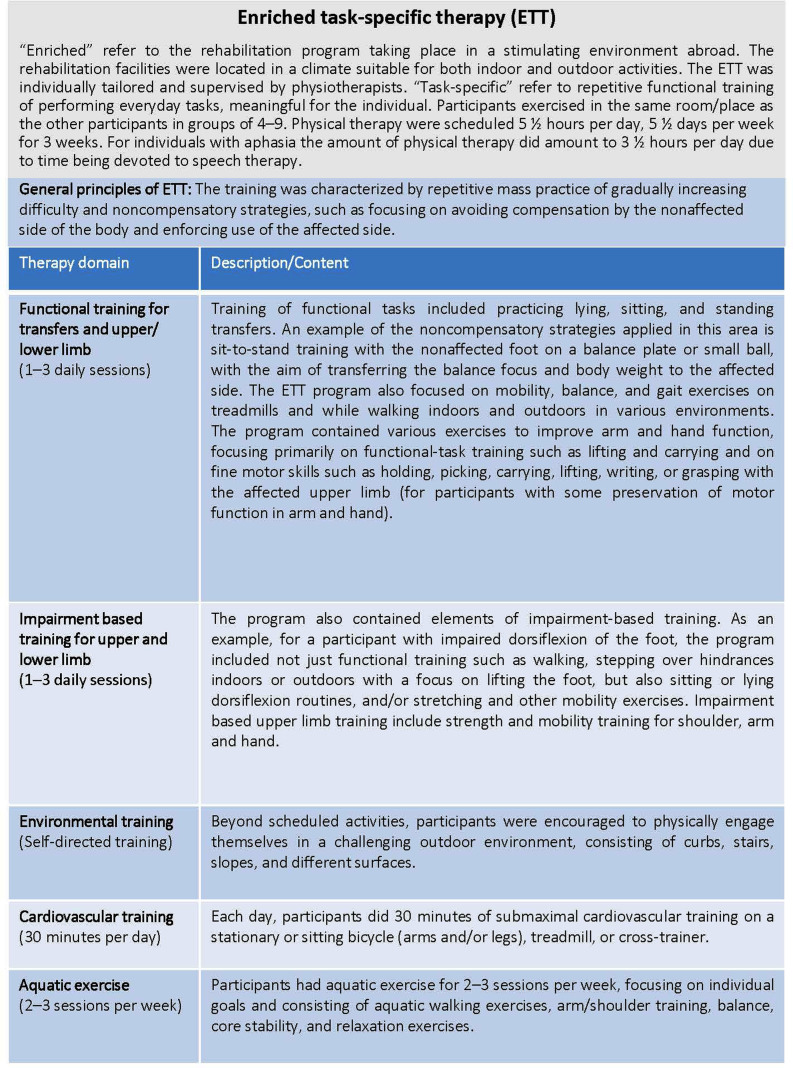
Motor components of ETT (enriched task-specific therapy).

The ETT program included a task-specific exercise, socialization, and sensory and cognitive stimulation at a rehabilitation facility located in Spain. The basics of task-specific training are the repetitive goal-directed practice of functional tasks that are meaningful for the individual. The intervention was based on non-compensatory strategies (Wolf et al., 2006; Kwakkel, Veerbeek, van Wegen, & Wolf, 2015). The various components of the enriched environment have previously been described in detail (S. Vive, Bunketorp-Kall, et al., 2020; S. Vive, Af Geijerstam, et al., 2020; S Vive et al., 2021). Some examples of the ETT content are presented in Fig. 2.

### 2.3 Measures

#### 2.3.1 30-second chair stand test

To measure lower extremity strength and endurance, the 30-second chair stand test (30sCST) was used (Jones, Rikli, & Beam, 1999). From the sitting position, the subject stands completely up and then sits completely back down, which is equal to one stand. The test measures the number of complete chair stands in 30 seconds. The test was performed three weeks before, directly before and after, and six months after the intervention. The test have shown excellent test-retest, interrater reliability and criterion validity (Jones et al., 1999). The range of number of chair stands in the age between 60– 89 is 8– 19 (Rikli & Jones, 1999).

### 2.4 Loading of limbs during STS using force plates

**Fig. 3a:**
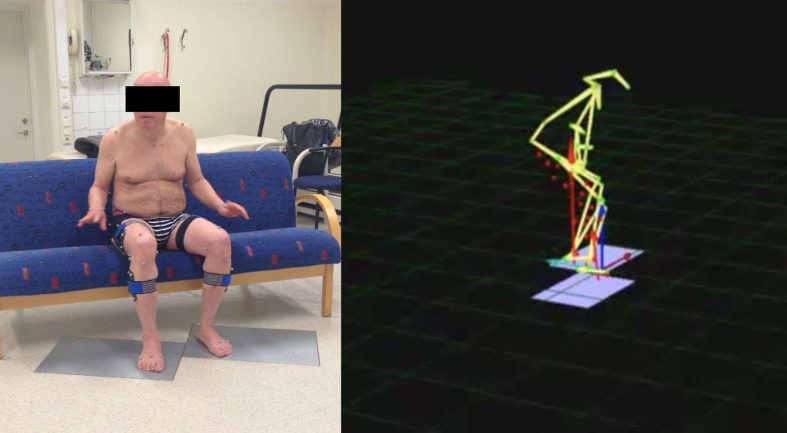
A photo of one subject on the test sofa, with his feet placed on the Kistler force plates. 3b. A 3D model of the subject in Visual 3D.

**Fig. 4:**
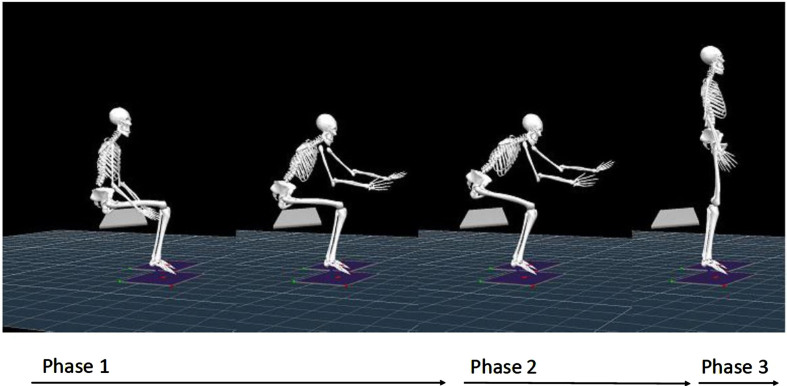
Illustration of the STS defined phases. *Phase 1*: the preparatory phase, defined as the onset of feet touching the force plate and lasting until seat-off. *Phase 2*: the extension phase, which lasts from seat-off (3) until the Thorax (Th) 2marker reaches the highest point (4). *Phase 3*: the stabilizing phase, at which the Th2marker reaches the highest point until stable standing at the end of the measurement.

The STS patterns and components were analyzed with an instrumental motion capture system (Qualisys, Göteborg, Sweden) combined with force plates at the Orthopaedic Research Unit at the Sahlgrenska University Hospital, Gothenburg, Sweden. The method is well described, reliable and validated in several studies (Tranberg, Saari, Zügner, & Kärrholm, 2011; Zügner, Tranberg, Lisovskaja, Shareghi, & Kärrholm, 2017; Zügner, Tranberg, Lisovskaja, & Kärrholm, 2018). The subject was asked to wear shorts or underwear and was instructed to stand up from a wooden and textile sofa (a) with no further instruction and (b) with an additional instruction to put as much weight as possible on the affected lower limb (Fig. 1a). A total of 3– 10 trials were registered for each condition (a and b). At the start of each measurement, the subjects placed their feet on two force plates (Kistler, Fig. 1a) that were integrated into the floor. Foot position and hand use were controlled. Spatiotemporal variables, kinematics, and kinetics were analyzed with the Visual 3D software (C-Motion, Germatown, MD, USA) (Fig. 1b) (Bell & Brand, 1989; Bell, Pedersen, & Brand, 1990; Weidow, Tranberg, Saari, & Karrholm, 2006; Tranberg et al., 2011; Zügner et al., 2017; Zügner et al., 2018). The GRF was measured for both the affected and unaffected lower limbs in three dimensions, and the vertical component of the vector was extracted and used for analyses. Figure 3a shows the setup and Fig. 3b the 3D model in Visual 3D. In this study, the same definitions of events (T_x_) and phases (1, 2, and 3; Fig. 4) of STS movement as previously presented were used (Fig. 4) (Vander Linden et al., 1994).

### 2.5 Statistics

Data from the 30sCST was analysed with the Wilcoxon signed-rank test. The data was analyzed according to the intention-to-treat (ITT) principle. Missing data was replaced with thelast-observation-carried-forward approach. The outcome of ETT was analyzed as the change from the second baseline assessment to the assessment immediately after ETT or follow-up. The kinetic data was normalised to 100% for comparisons among different trials. The maximum vertical component for every trial during a phase was extracted, and the mean from each phase was calculated. The ratio between limbs was calculated by taking the affected limb’s mean of maximum vertical force during a phase divided by the corresponding value of the unaffected limb. Descriptive statistics were used and described for each timepoint and for each STS phase in all three subjects. To determine whether the outcome variables approximated a normal distribution, quantile– quantile plots and histograms as well as skewness and kurtosis were used. Since the normal distributionplots were found satisfactory, a semi-statistical approach was used, the two-standard deviation band (TSDB) method, in which significant changes are identified by a visual inspection of graphs. Treatment effects were considered to be obvious from visual inspection of the data (Ottenbacher, 1986). Horizontal bands representing±2 SD of the baseline data (Phase A1) were drawn on the graphs. A change was defined as significant if at least two successive observations in one phase were outside the±2 SD range from the previous phase (Kazdin, 2019). The values are reported as mean±SD.

## 3 Results

The demographic and clinical characteristics of the study subjects are presented in Table 2. Figure 1 shows a flowchart of the study as well as the enrollment and assessment of the participants.

**Table 2:** Demographic and clinical characteristics of the study subjects

Subject, nr	Age (y)	Gender (M/F)	Months since stroke onset, mean (SD) median [min;max]	MRS (0– 6), Mean grade, mean (SD), median [min;max]	Right hemispheric stroke / Left hemispheric
N = 41	59.6 (13.9)	33/8	35.5 (29.5)	3.4 (0.7)	18 (44%)
Whole study group	64 [22;84]		26 [6;130]	3.0 [2;4]	23 (56%)
N = 1, Subject 1	62	M	32	2	L
N = 1, Subject 2	65	M	88	3	R
N = 1, Subject 3	57	M	14	3	L

### 3.1 30-second chair stand test

After the baseline period, within-group repeated-measure analyses revealed a small yet significant improvement with respect to strength and endurance as measured with the 30sCST (from 5.6±4.5 to 6.1±4.9, *p* = 0.042, mean change 0.6±1.8). At post-intervention (ETT), an even larger significant increase in STS performance was seen (from 6.1±4.9 to 8.2±5.4, *p* < 0.001, mean change 2.1±2.9). The enhanced STS performance was sustained at the six-month follow-up for the study group (8.1±5.3, *p* < 0.001, mean change 2.0±2.4) (*n* = 41, ITT = 5, PP – *n* = 36). On the individual level, the three subjects included in the in-depth study increased their scores on the 30sCST by five, three, and two, respectively, immediately after the intervention and by five, two, and zero, respectively, at the long-time follow-up.

### 3.2 Loading of the affected limb during the STS performance when administered without instructions

**Fig. 5:**
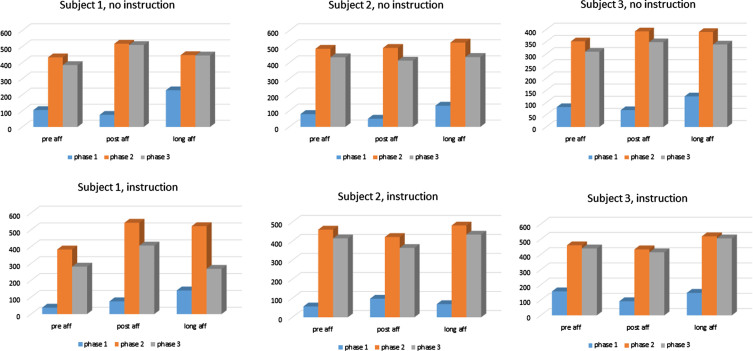
Mean of maximum F-vertical for the affected side during Phases 1, 2, and 3, with and without instructions, at pre-, post-, and long-term follow-up for all three subjects.

The results from the in-depth analyses of the mean vertical GRF of the maximum peak during all three phases are presented in Fig. 5. When the STS exercise was performed without specific instructions, the mean loading of the affected limb during the extension phase (Phase 2) for Subject 1 increased by 19.5% (+84.8 N) from pre-to post-intervention. Six months post-intervention, the mean loading was reduced and approached the pre-intervention value (+13.7 N/+3.2%). The ratio between loading of the affected and non-affected limbs during Phase 2 was similar at all three assessment time points (1.08, 1.08, and 1.09).

For Subject 2, the increase in the mean loading of the affected limb during the extension phase from pre-topost-intervention was negligible (+5.1 N/+1.1%). Six months post-ETT, the mean loading of the affected limb during the extension phase was 38.3 N (+7.9%) higher as compared to pre-intervention. The ratio between loading of the affected and non-affected limbs during the extension phase at pre-and post-intervention was 0.54 and 0.58, respectively, and 0.46 at the six-month follow-up.

For Subject 3, the mean loading of the affected limb during the extension phase increased by11.7% (+41.1 N) from pre-to post-intervention and 9.9% (+38.6 N) from before to the six-month follow-up. The ratio between loading of the affected and non-affected limbs during Phase 2 at pre-and post-intervention was 0.90 and 1.01, respectively, and 1.08 at the six-month follow-up.

### 3.3 Loading of the affected limb during the STS performance when administered with instructions

With the instruction to put the maximum load on the affected limb, the mean loading during the preparatory phase for Subject 1 was almost doubled post-intervention (+97.9% /+37.4 N). The loading during the extension phase increased by 41.3% (+158.4 N) from pre-to post-intervention. At six months, the loading during the preparatory phase had increased further (+267% /+102.0 N). At six months, the increase during the extension phase was still present yet somewhat reduced (+138.2 N/+36.1%). The ratio between loading of the affected and non-affected limbs during Phase 2 increased from 0.67 at pre-intervention to 0.99 at post-intervention and 0.84 six months later.

For Subject 2, the loading of the affected limb during the preparatory phase increased by 72.4% (+41.0 N) with instructions, whereas the loading during the extension phase was reduced (– 38.7 N/– 8.4%) as compared to before the intervention. Six months post-ETT, the loading was 22% higher (+12.5 N) during the preparatory phase as compared to before the ETT and 4.7% higher (+21.7 N) during the extension phase as compared to pre-intervention. The ratio between loading of the affected and non-affected limbs during Phase 2 at pre-and post-intervention was 1.35 and 0.89, respectively, and 1.02 at the six-month follow-up.

For Subject 3, the mean loading of the affected limb during both the preparatory phase (– 64.8 N/– 41.1%) and the extension phase (– 26.9 N/– 5.8%) was reduced as compared to the baseline. Six months after the ETT, the loading was 6.2% lower (– 9.7 N) during the preparatory phase but 12.8% (+59.1 N) higher during the extension phase as compared to pre-intervention. The ratio between loading of the affected and non-affected limbs during Phase 2 at pre-and post-intervention was 1.39 and 1.28, respectively, and 1.44 at the six-month follow-up.

### 3.4 Two-standard deviation band method

**Fig. 6:**
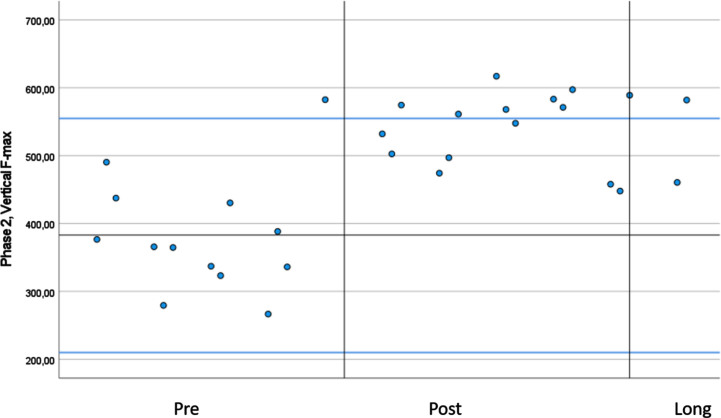
The vertical GRF (ground reaction force) at the three test occasions for Subject 1 using the 2SDbands in blue and the middle line mean. The plots represent the loading of the affected lower limb during the extension phase in the STS test performed with instructions.

In Table 3, the mean scores of the maximum F-vertical for the affected side during the extension phase (Phase 2) of the STS test performed without instructions are shown. Using the TSDB, only Subject 1 had a significant change in vertical F-max after the intervention, with more than two consecutive measures larger than +2SD from Phase A1 (Fig. 6). The increase was not sustained at the six-month follow-up, as indicated by one single measurement remaining over the 2SD band at that time point. For Subjects 2 and 3, the vertical F-max for the affected limb remained unchanged during the study, independent of the instructions given to the participants.

**Table 3:** Maximum ground reaction force during the extension phase, with instruction, affected side

Vertical F-max with instruction phase 2, affected side	Mean Pre (SD)	+2SD, – 2SD	Mean Post (SD)	Mean Long (SD)
Subject 1	383.0 (86.3)	555.6	541.4 (53.3)	521.3 (85.8)
		210.4
Subject 2	475.5 (56.5)	572.8	461.9 (42.8)	503.6 (34.8)
		141.6
Subject 3	460.4 (112.9)	686,2	433.4 (99.8)	519.5 (15.8)
		234.6

STS movement performed at all three test occasions with the instruction to put the maximum load on the affected lower limb. Pre: pre-intervention, Post: post-intervention; Long: 6 months after the intervention. Vertical F-max: The maximum peak of the vertical component of the ground reaction force; SD: standard deviation.

## 4 Discussion

This study demonstrates that an ETT program may provide long-term benefits in STS performance as measured by the 30sCST in the chronic phase after stroke. Considering that the primary analyses of study data from the first to the second baseline assessment did not demonstrate any changes across several measures apart from a decrease in health-related QoL (S. Vive, Af Geijerstam, et al., 2020), this suggests that the participants were in a stable phase after stroke. The small improvement revealed at the second baseline assessment was most likely caused by a systematic measurement error given the fact that the design of the chair used for the 30sCST in Spain was not identical to the chair used at the first baseline assessment in Sweden. This may have impacted the test results by systematically yielding better test scores. The improvement demonstrated at the end of the ETT program was more than threefold the increase at the second baseline assessment, speaking in favor of an intervention effect.

There is no data available on the minimal clinically important difference (MCID) of the 30sCST in a population of individuals with chronic stroke. However, in community-dwelling individuals with hip osteoarthritis, the MCID of the 30sCST was found to be 2.0, 2.6, and 2.1 repetitions depending on the method used for analyses (Wright, Cook, Baxter, Dockerty, & Abbott, 2011). In the osteoarthritis study, the number of STS repetitions at the baseline was 10.1 (±4.4), whereas in our study, the score at the baseline was 5.6 (±4.5), thus indicative of the stroke cohort having greater functional impairment as compared to the osteoarthritis group. This might suggest the short- and long-term improvements of 2.1 and 2.0, respectively, being clinically meaningful for the individuals. Conversely, even though the change of 0.6 during the baseline period was statistically significant, it is likely too small to be considered clinically relevant.

The in-depth weight-bearing STS analyses demonstrated a variety among the three subjects, where only one (Subject 1) seemed to consistently put a higher load on the affected leg after the intervention, especially when directing the subjects’ attention and effort toward the affected limb. By applying the TSDB method to the data, the increase from pre-to post-intervention (without instructions) for Subject 1 was shown to be significant. An increase was still present at the six-month follow-up for Subject 1, though not significant according to the TSDB. Of the three subjects included in the in-depth study, Subject 1 had the largest increase in the 30sCST (+5) from pre-to post-intervention. This improvement may be mediated by the more symmetrical STS performance and increased ability of generating force of the affected lower-limb muscles during the STS action (Fowler & Carr, 1996; Dean, Richards, & Malouin, 2000). For the other two subjects, slightly different strategies were seen during the STS movement after the intervention. For Subject 2, when receiving instructions to put as much load as possibleon the affected limb, he was able to increase the load during the preparatory phase, but during lift-off, where the extension phase (2) started and the subject was to defeat the center of mass against gravity, the load shifted, and no increase was seen. Without the instruction, negligible or small increases in the loading patterns of the affected limb were seen directly and six months after the intervention for Subject 2. At post-intervention and six months, the 30sCST score for Subject 2 was slightly higher than the baseline (+3 and +2, respectively), which may be the result of compensatory movement strategies. For Subject 3, performing the STS task without instructions resulted in a more symmetrical pattern after the intervention. However, when adding the therapeutic instruction to the task, the increased load was not different from before the intervention.

Performance on the STS test has often been seen as an indicator or proxy measure for lower limb strength in older people (Yee et al., 2021). However, previous studies have found that balance and mobility are also important determinants of STS performance after stroke (Janssen, Bussmann, & Stam, 2002). Researchers have additionally investigated the association between STS performance and physical capacity (Azharuddin & Zia, 2021). It has been argued that individuals with slight disability might be more responsive to interventions aiming to improve movement quality than those with higher disability. The fact that Subject 1 was judged to have an MRS score of 2 (slight disability), whereas Subjects 2 and 3 were graded MRS 3 (moderate disability), may suggest that Subject 1 was more responsive to the ETT program. Since individuals with stroke-related hemi paresis bear most of their body weight on their unaffected limb, leading to learned non-use syndrome, (Roy et al., 2006) one should focus on directing the individuals’ attention and effort toward the affected limb with the aim to reverse this tendency and achieve more symmetrical movement (Taub et al., 1993). We therefore performed the kinetic analyses of the STS task with and without instructions and observed that all three subjects were able to put more weight on their affected side in one or more phases of the movement when receiving such instructions. Previous studies have described the weight distribution during STS movement for individuals with stroke and shown that during a habitually performed STS exercise, they put 33.5% of their bodyweight on the affected side, and with instructions to put symmetrical weight, they put 44.4% of their bodyweight on the affected side (Engardt & Olsson, 1992). Other studies have reported loading of the affected side during STS movement to range between 24% and 37% of the body weight (Engardt, 1994; Cheng, Wu, Liaw, Wong, & Tang, 2001). In our study, the individuals’ ratio of loading the affected leg versus the non-affected limb varied between 0.54 and 1.09 during the habitually performed extension phase.

### 4.1 Limitations

The study has acknowledged limitations, (S. Vive, Af Geijerstam, et al., 2020) one being the within-subject, repeated-measure design with no comparison group to account for the effects of participating in a rehabilitation program of any type. Another limitation is that the assessment site was not identical across all assessment time points in the study, which may have introduced systematic measurement errors such as differences in measurement equipment. For the in-depth analysis of STS performance, we used a SSED, and the TSDB method was used to determine whether any significant changes occurred as a result of the intervention (S. Vive, Af Geijerstam, et al., 2020). However, the sample size was small, and the TSDB method has a high agreement with the C statistic method (Nourbakhsh & Ottenbacher, 1994) but is nevertheless sensitive to extreme values; one outlier can largely influence the variance in a small set of data and produce misleading results. Yet another limitation of this study is that the maximum vertical force component for every trial during a phase was extracted, which produced a large range of forces. To avoid one single outlier measurement affecting the results, a mean of several trials was calculated. However, the fact that just the peak force was described during a phase and not the force production during a whole phase might limit the result to one parameter with no information on the complexity of the movement.

## 5 Conclusion

The group-level analyses in this study support the efficacy of ETT in producing long-term gains in STS performance. The in-depth assessment of STS kinetics revealed that one of three subjects reached significant improvement in weight-bearing patterns during STS performance after the intervention, suggesting that weight-bearing strategies could be one of several factors that contribute to improvements in such performance. Further, the STS movement differed considerably with the subject’s intent on the movement, such as loading the affected leg. Larger and controlled studies with laboratory measures of weight-bearing during STS performance are needed to further investigate the effects of specific training protocols as well as to increase the understanding of factors affecting STS performance in the chronic phase after stroke.

## Conflict of interest

The authors declare no conflicts of interest.

## Ethics statement

This study followed the recommendations provided by the Single-Case Reporting Guideline in Behavioral Interventions (SCRIBE). The study was approved by the Regional Ethical Review Board in Gothenburg, Sweden (Ref. No. 549-12) and done in accordance with relevant ethical guidelines. All participants received detailed study information, signed a written informed consent form, and were told that they could withdraw from the study at any time. The study complies with the Declaration of Helsinki.

## Funding

The authors would like to thank the following funding agencies for supporting the study: the AinaWallström’s and Mary-Ann Sjöblom’s Foundation, Peter Eriksson Foundation, the Swedish state under the agreement between the Swedish government and the county councils, the ALF-agreement (725241), Promobilia foundation, the Swedish Stroke Association, Rune and Ulla Amlöv’s foundation, Renée Eander Foundation.
